# Supplementary material for: Stratification of hospitalized COVID-19 patients into clinical severity progression groups by immuno-phenotyping and machine learning
Source: Nat Commun. 2022 Feb 17;13:915. doi: 10.1038/s41467-022-28621-0 (PMC8854670; doi:10.1038/s41467-022-28621-0)
Supplement: Supplementary file 1 — Supplementary Information [file 41467_2022_28621_MOESM1_ESM.pdf]

Stratification of hospitalised COVID-19 patients into clinical severity progression groups by immuno-phenotyping and machine learning

Yvonne M. Mueller[1], Thijs J. Schrama[1], Rik Ruijten[1], Marco W.J. Schreurs[1], Dwin G.B. Grashof[1], Harmen J. G. van de Werken[1,2], Giovanna Jona Lasinio[3], Daniel Álvarez-Sierra[4], Caoimhe H. Kiernan[1], Melisa D. Castro Eiro[1], Marjan van Meurs[1], Inge Brouwers-Haspels[1], Manzhi Zhao[1], Ling Li[1], Harm de Wit[1], Christos A. Ouzounis[5,6], Merel E. P. Wilmsen[1], Tessa M. Alofs[1], Danique A. Laport[1], Tamara van Wees[1], Geoffrey Kraker[7], Maria C. Jaimes[7], Sebastiaan Van Bockstael[7], Manuel Hernández-González[4], Casper Rokx[8], Bart J.A. Rijnders[8], Ricardo Pujol-Borrell[4,9], Peter D. Katsikis[1]

Supplementary Information

Supplementary Figure 1
Supplementary Figure 2
Supplementary Figure 3
Supplementary Figure 4
Supplementary Table 1
Supplementary Table 2
Supplementary Table 3

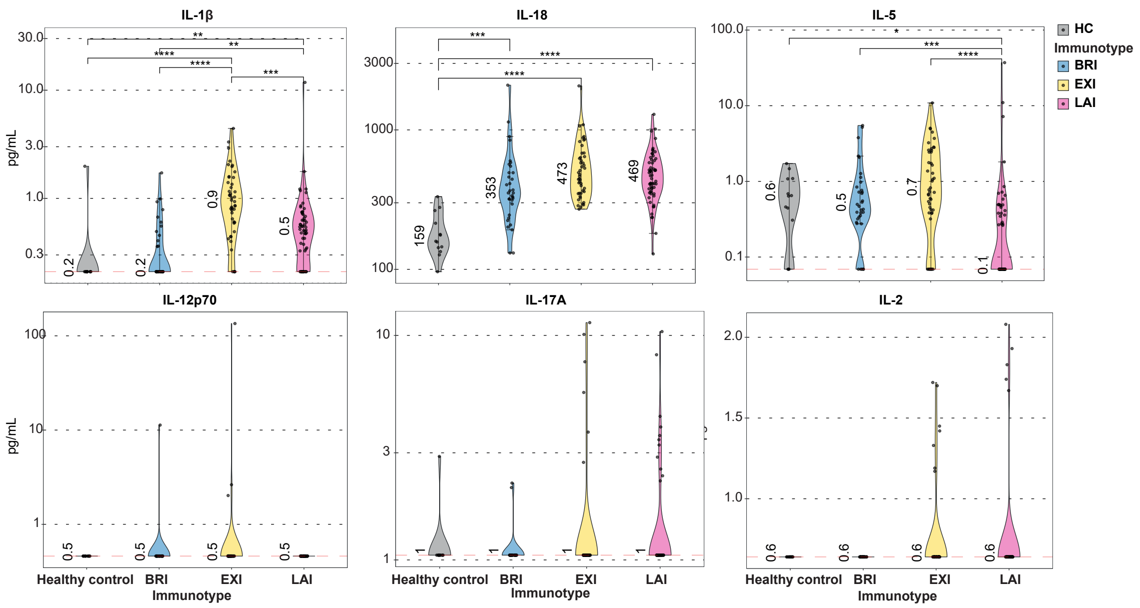

Supplementary figure 1: Violin plots depicting serum levels for IL-1β, IL-18, IL-5, IL-12, IL-17A and IL-2 in the patients of different immunotypes and healthy controls. Data shown are from BRI (n=33), EXI (n=46) and LAI (n=59) patients, and healthy controls (n=14). Median values are in numbers and whisjers indicate4 the 1.5 x IQR value. Two sided Wilcoxon rank-sum tests with Bonferroni correction were applied for each measurement. Red dashed line indicates assay Limit of Detection. *p < 0.05, **p < 0.01, ***p < 0.001, and ****p < 0.0001.

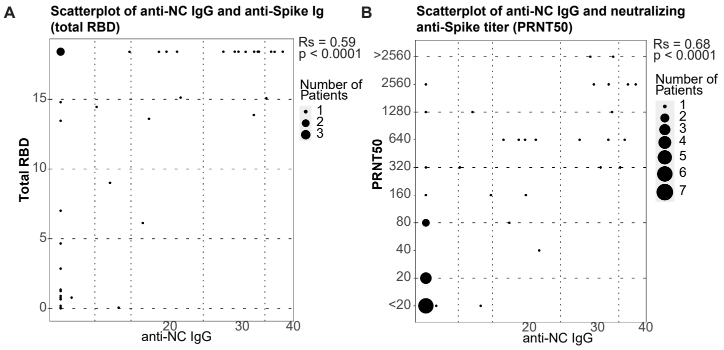

Supplementary figure 2: Anti-NC antibodies correlate with anti-Spike antibodies and neutralization titers. Scatterplots shown between A) anti-NC IgG and anti-Spike RDB Ig, and B) anti-NC IgG and neutralizing anti-SARS-CoV-2 titer. Spearman's rank correlation coefficient and p values are indicated.

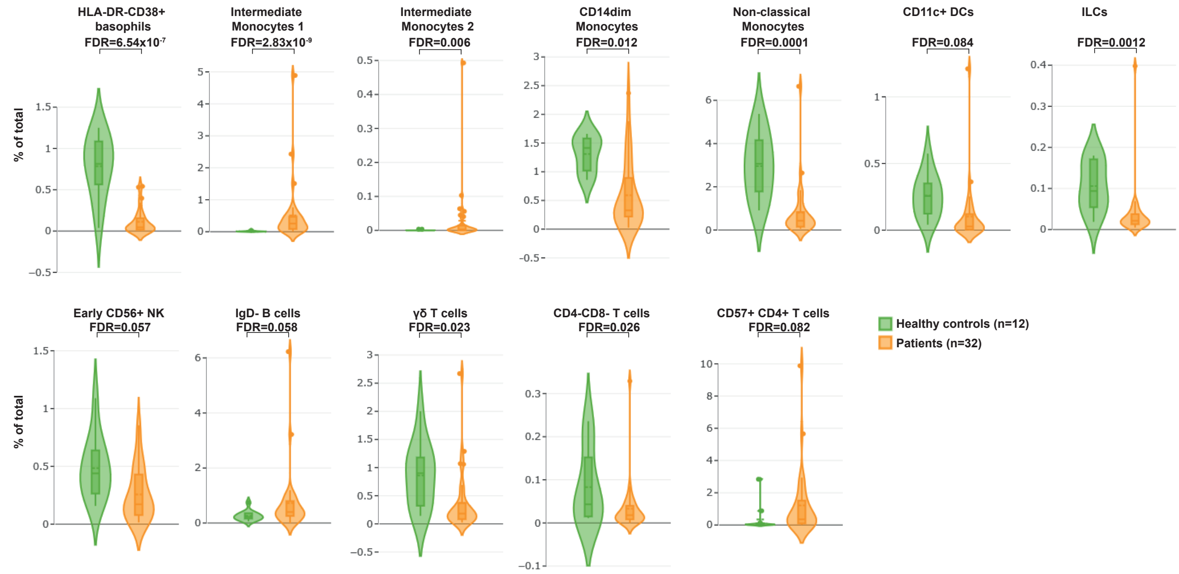

Supplementary figure 3: COVID-19 patient (n=35) and healthy control (n=12) peripheral blood cellular populations that were identified by high dimensional 40-color flow cytometry to be significantly different are shown (FDR<0.1). Violin plots show the range of differentially expressed blood populations and depict the percentage of cells in the total population. The violin shapes were defined by kernel density estimation, the boxes define the Q1 to Q3 range while box whiskers indicate min/max. The mean and median are indicated by dotted and solid middle lines, respectively.

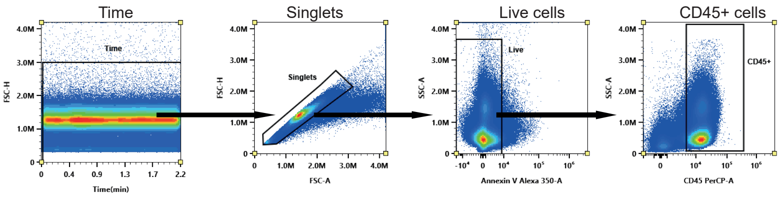

Supplementary figure 4: Clean-up of flow cytometry data. PBMC were stained with a 40-color antibody panel, data was collected using a 5 laser Aurora spectral flow cytometer. Clean up of flow cytometry data was performed in SpectroFlo (version 2.2.0). The gating strategy is depicted. The cleaned flow data was imported into OMIQ data analysis software for further unsupervised analysis based on phenotypical surface markers without any additional 2D gating. Subsequently, the workflow included running flowCut to check for changes in channels over acquisition time, UMAP for dimensionality reduction, flowSOM for clustering, and edgeR for statistical inference.

Supplementary Table 1. Univariate and multivariate analysis with Gender, Age and DFSO as additional variables									
	BRI vs EXI			BRI vs LAI			EXI vs LAI		
Variable	Wilcoxon test	Univariant model Coefficient (CI/significance)	Multivariate model Coefficient (CI/significance)	Wilcoxon test	Univariant model Coefficient (CI/significance)	Multivariate model Coefficient (CI/significance)	Wilcoxon test	Univariant model Coefficient (CI/significance)	Multivariate model Coefficient (CI/significance)
IL-6	****	2.17 (1.41-2.93/****)	2.07 (1.25-2.9/****)	****	1.56 (0.86-2.26/****)	1.53 (0.77-2.29/****)	*	-0.61 (-0.99--0.23/**)	-0.54 (-0.97--0.11/*)
TNFα	****	4.73 (2.9-6.57/****)	4.47 (2.47-6.46/****)	*	2.02 (0.53-3.52/**)	1.79 (0.13-3.46/*)	***	-2.72 (-4.12--1.32/***)	-2.67 (-4.2--1.15/***)
CCL2	****	2.52 (1.47-3.57/****)	2.02 (0.93-3.12/***)	**	1.59 (0.64-2.54/**)	1.29 (0.28-2.31/*)	*	-0.93 (-1.61--0.26/**)	-0.73 (-1.45--0.01/*)
IL-8	****	1.91 (1.15-2.68/****)	2.32 (1.42-3.23/****)	0.18 ns	0.63 (0-1.27/ns)	**0.78 (0.04-1.52/*)**	****	-1.28 (-1.86--0.7/****)	-1.54 (-2.22--0.86/****)
IL-10	****	2.7 (1.64-3.76/****)	2.51 (1.37-3.64/****)	****	3.07 (1.99-4.15/****)	2.95 (1.8-4.09/****)	0.98 ns	0.37 (-0.21-0.95/ns)	0.44 (-0.21-1.09/ns)
IFNα	0.186 ns	**0.59 (0.05-1.12/*)**	**0.99 (0.28-1.7/**)**	****	1.81 (1.2-2.42/****)	2.15 (1.37-2.94/****)	****	1.22 (0.8-1.64/****)	1.17 (0.72-1.61/****)
IFNγ	0.132 ns	**0.43 (0.08-0.78/*)**	**0.45 (0.05-0.84/*)**	**	0.64 (0.28-0.99/***)	0.68 (0.28-1.07/***)	1 ns	0.21 (-0.08-0.5/ns)	0.23 (-0.1-0.55/ns)
TGFβ1	1 ns	-0.35 (-1.46-0.76/ns)	0.08 (-1.24-1.4/ns)	**	-1.67 (-2.74--0.59/**)	-1.3 (-2.49--0.11/*)	**	-1.32 (-2.23--0.41/**)	-1.38 (-2.45--0.31/*)
IgM	0.134 ns	**-0.89 (-1.66--0.12/*)**	**-1.15 (-2.04--0.25/*)**	****	-10.08 (-19.29--0.87/*)	-8.38 (-16.36--0.41/*)	****	**-9.19 (-18.38-0.01/ns)**	**-7.24 (-15.21-0.72/ns)**
IgG	1 ns	-0.56 (-1.38-0.27/ns)	-0.68 (-1.68-0.32/ns)	****	-4.15 (-5.65--2.64/****)	-4.54 (-6.23--2.84/****)	****	-3.59 (-5.03--2.16/****)	-3.85 (-5.43--2.28/****)
IgA	1 ns	0.06 (-0.51-0.63/ns)	0.14 (-0.54-0.81/ns)	****	-2.78 (-3.78--1.78/****)	-2.77 (-3.82--1.73/****)	****	-2.84 (-3.82--1.86/****)	-2.91 (-3.93--1.9/****)
Peak WHO	****	2.33 (1.41-3.26/****)	2.21 (1.16-3.25/****)	****	1.81 (0.98-2.64/****)	1.93 (0.97-2.88/****)	1 ns	-0.52 (-1.21-0.17/ns)	-0.28 (-1.05-0.49/ns)
Entry WHO	***	2.21 (1.13-3.28/****)	2.16 (0.92-3.4/***)	0.24 ns	0.94 (-0.01-1.89/ns)	1.03 (-0.05-2.11/ns)	*	-1.27 (-2.14--0.39/**)	-1.13 (-2.12--0.14/*)
CRP	**	1.26 (0.58-1.94/***)	1.2 (0.43-1.97/**)	1 ns	0.33 (-0.18-0.85/ns)	0.36 (-0.24-0.96/ns)	**	-0.93 (-1.55--0.31/**)	-0.84 (-1.5--0.18/*)
d-dimer	0.066 ns	**0.73 (0.2-1.26/**)**	**0.74 (0.15-1.34/*)**	0.9 ns	-0.25 (-0.77-0.27/ns)	-0.25 (-0.86-0.36/ns)	**	-0.98 (-1.48--0.49/***)	-0.99 (-1.55--0.44/***)
ferritin	***	1.07 (0.49-1.65/***)	1.17 (0.41-1.93/**)	0.54 ns	**0.5 (0.03-0.96/*)**	0.61 (-0.04-1.25/ns)	*	-0.57 (-1.09--0.05/*)	**-0.56 (-1.16-0.04/ns)**
LDH	***	2.83 (1.29-4.38/***)	3.01 (1.23-4.8/***)	0.42 ns	**1.69 (0.24-3.14/*)**	**2.08 (0.4-3.77/*)**	*	-1.15 (-2.22--0.08/*)	**-0.93 (-2.04-0.18/ns)**
neutrophils	*	1.28 (0.3-2.25/*)	1.18 (0.05-2.31/*)	0.7 ns	-0.74 (-1.66-0.17/ns)	-0.59 (-1.61-0.43/ns)	****	-2.02 (-2.97--1.06/****)	-1.77 (-2.78--0.76/***)
lymphocytes	0.26 ns	-0.97 (-2.09-0.14/ns)	-0.5 (-1.78-0.77/ns)	0.2 ns	**-1.11 (-2.19--0.04/*)**	-0.59 (-1.79-0.62/ns)	1 ns	-0.14 (-1.04-0.76/ns)	-0.09 (-1.11-0.93/ns)
thrombocytes	1 ns	-0.25 (-1.31-0.81/ns)	0.26 (-1.04-1.56/ns)	***	-2.25 (-3.42--1.07/***)	-1.94 (-3.24--0.64/**)	**	-1.99 (-3.07--0.92/***)	-2.2 (-3.42--0.98/***)
IL-1β	****	2.01 (1.23-2.78/****)	2.15 (1.3-3.01/****)	*	0.99 (0.33-1.66/**)	1.1 (0.37-1.83/**)	***	-1.01 (-1.59--0.44/***)	-1.06 (-1.66--0.45/***)
IL-18	0.092 ns	**1.33 (0.29-2.37/*)**	1 (-0.13-2.13/ns)	0.174 ns	0.86 (-0.12-1.83/ns)	0.83 (-0.21-1.87/ns)	1 ns	-0.47 (-1.31-0.36/ns)	-0.17 (-1.1-0.75/ns)
IL-5	0.52 ns	0.19 (-0.13-0.51/ns)	0.11 (-0.25-0.47/ns)	***	-0.58 (-0.92--0.24/***)	-0.56 (-0.93--0.19/**)	****	-0.78 (-1.11--0.44/****)	-0.67 (-1.01--0.34/****)
IL-12p70	1 ns	0.19 (-0.5-0.87/ns)	-0.33 (-1.46-0.8/ns)	1 ns	-4.57 (-45.06-35.91/ns)	**-4.83 (-6.48--3.18/****)**	0.3 ns	-4.41 (-35.26-26.43/ns)	**-5.93 (-7.59--4.28/****)**
IL-17A	1 ns	1.28 (-0.33-2.88/ns)	1.16 (-0.59-2.9/ns)	0.44 ns	1.31 (-0.28-2.89/ns)	1.39 (-0.31-3.08/ns)	1 ns	0.03 (-0.65-0.71/ns)	0.23 (-0.54-0.99/ns)
IL-2	1 ns	1.74 (-0.48-3.97/ns)	1.24 (-1.24-3.72/ns)	1 ns	1.49 (-0.72-3.71/ns)	1.31 (-1.15-3.77/ns)	1 ns	-0.25 (-1.42-0.92/ns)	0.07 (-1.21-1.35/ns)

Supplementary Table 1. Univariate and multivariate regression analysis with Gender, Age and DFSO as additional variables. Wilcoxon rank-sum statistical tests with Bonferroni correction was applied on six comparisons (including Healthy controls, comparisons not shown). A uni- and multiple variable analyses with Gender, Age and ln(DFSO+1) as additional variables were applied. We estimated logistic multinomial models, built using one log-transformed variable at a time. Models' coefficients with their 95% Confidence interval and significance score are reported. Values in bold indicate differences in statistical significance between Wilcoxon test, Uni- and Multivariate analyses. Data included are from BRI (n=33), EXI (n=46) and LAI (n=59) patients. For the Multivariate analysis patients with non-available DFSO were excluded. *p < 0.05, **p < 0.01, ***p < 0.001, and ****p < 0.0001.

Supplementary Table 2: High dimensional flow cytometry comparisons and differentially expressed cellular populations

COVID-19 Patients : Healthy controls ratio			
**Cell population**	**logFC**	**P-Value**	**FDR**
Plasmablasts	4.34	1.23E-14	4.19E-13
Intermediate Monocytes 1	5.31	1.67E-10	2.83E-09
HLA-DR- CD38+ Basophils	-2.78	5.77E-08	6.54E-07
CD123+ pDC	-1.67	3.69E-06	3.13E-05
Non-classical Monocytes	-1.88	1.84E-05	0.0001
ILCs	-1.49	0.0003	0.0012
Intermediate Monocytes 2	4.77	0.001	0.006
$CD14^{dim}$ Monocytes	-1.14	0.003	0.012
γδ T cells	-1.20	0.007	0.023
CD4- CD8- T cells	-1.32	0.008	0.026
Early $CD56^{bright}$ NK cells	-0.91	0.02	0.057
IgD- CD27- B Cells	1.44	0.02	0.058
CD57+ CD4+ T cells	1.83	0.03	0.082
CD11c+ DCs	-1.33	0.03	0.084
CD38+ $CD56^{dim}$ NK cells	0.83	0.04	0.103

EXI:BRI immunotype ratio			
**Cell population**	**logFC**	**P-Value**	**FDR**
Intermediate Monocytes 2	2.97	0.008	0.26

LAI:BRI immunotype ratio			
**Cell population**	**logFC**	**P-Value**	**FDR**
Intermediate Monocytes 1	2.19	0.004	0.14
CD8+ NKT cells	-2.249	0.009	0.16
CD4- CD8- T cells	1.68	0.024	0.18
ILCs	1.39	0.032	0.18
Early $CD56^{bright}$ NK cells	1.18	0.035	0.18
CD38+ $CD56^{dim}$ NK cells	1.12	0.035	0.18
HLA-DR- CD38+ Basophils	-1.39	0.038	0.18

LAI:EXI immunotype ratio			
**Cell population**	**logFC**	**P-Value**	**FDR**
Intermediate Monocytes 2	-4.26	8.92E-05	0.003
CD4+ CD8+ T cells	-1.76	0.011	0.18
CD45RA- γδ T cells	-1.46	0.029	0.25
CD38+ $CD56^{dim}$ NK cells	1.39	0.036	0.25
IgD- CD27- B Cells	-1.57	0.037	0.25

Supplementary Table 2. High dimensional flow cytometry identified differentially expressed cellular populations. The peripheral blood cellular populations identified by high dimensional 40-color flow cytometry from indicated pairwise comparisons are shown. Comparisons between COVID-19 patients (n=32) and healthy controls (n=12), and pairwise comparisons of immunotypes (BRI n=9, EXI n=6 and LAI n=17) shown. Positive logFC indicates numerator of ratio is increased while negative values indicate a decrease. LogFC indicate log2 fold change. For statistical analysis, two-sided edgeR (adapted Fisher's exact test) was used, p value is uncorrected. The p value given by FDR is corrected using the Benjamini and Hochberg method for multiple testing correction. Comparisons with FDR<0.1 are considered to be significantly different. All population comparisons with a p<0.05 are shown.

Supplementary Table 3. Exact p values for figures 2, S1, 3, 4A and 5.

Figure 2 p-values

Group comparisons	P adjusted values					
	IL-6	TNFa	CCL2	IL-8	IL-10	IFNa
HC vs BRI	7.2e-7	1.3e-2	0.46	1.3e-8	4 e- 7	4.1e- 1
HC vs EXI	1.2e-7	5.6e-7	0.0000019	1.2e-7	1.1e- 6	2.2e- 2
HC vs LAI	4.5e-8	5.7e-5	0.000029	7.7e-8	1.2e- 7	2 e- 7
BRI vs EXI	5 e-8	2.4e-7	0.0000038	2.1e-6	4.2e- 7	1.9e- 1
BRI vs LAI	8.5e-7	4.2e-2	0.0031	1.8e-1	1.4e-11	4 e-12
EXI vs LAI	2.4e-2	1.3e-4	0.034	2.6e-5	9.8e- 1	3.9e-10

Group comparisons	P adjusted values				
	IFNg	TGFb	IgM	IgG	IgA
HC vs BRI	0.0035	1	1.9e- 3	3 e- 5	7.5e- 6
HC vs EXI	0.00012	1	5.5e- 2	8.9e- 5	3.3e- 5
HC vs LAI	0.0000028	0.026	1 e+ 0	5.1e- 1	1.5e- 1
BRI vs EXI	0.13	1	1.3e- 1	1 e+ 0	1 e+ 0
BRI vs LAI	0.0014	0.0026	6.9e-10	1.7e-10	1.4e-10
EXI vs LAI	1	0.0014	1.2e- 5	4.3e- 9	6.6e-10

Supplementary Figure 1 p-values

Group comparisons	P adjusted values					
	IL-1b	IL-18	IL-5	IL-12p70	IL-17A	IL-2
HC vs BRI	4.3e-1	3.4e-4	1	1	1	1
HC vs EXI	6.2e-5	7.2e-7	1	1	1	0.71
HC vs LAI	1.3e-3	1.3e-6	0.047	1	1	1
BRI vs EXI	6.8e-7	9.2e-2	0.51	1	1	0.1
BRI vs LAI	9 e-3	1.7e-1	0.00048	0.94	0.44	0.45
EXI vs LAI	1.6e-4	1 e+0	0.0000019	0.24	1	1

Figure 3 p-values

Group comparisons	P adjusted values				
	WHO_Entry_D1	WHO_Peak	total_days_in_hospital	total_days_on_ICU	Age
BRI vs EXI	0.00027	1.2e-7	0.000088	0.000052	0.051
BRI vs LAI	0.12	3.6e-5	0.00016	0.00015	0.17
EXI vs LAI	0.0097	8.2e-1	0.46	0.83	1

Figure 4A p-values

Group comparisons	P adjusted values
	DFSO
BRI vs EXI	1
BRI vs LAI	0.0038
EXI vs LAI	0.016

Figure 5 p-values

Group comparisons	P adjusted values			
	CRP	d-Dimer	Ferritin	LDH
BRI vs EXI	0.0012	0.033	0.00019	0.00026
BRI vs LAI	0.85	0.45	0.27	0.21
EXI vs LAI	0.0041	0.00069	0.02	0.019

Group comparisons	P adjusted values			
	Neutrophils	Lymphocytes	Thrombocytes	Viral Loads
BRI vs EXI	0.01	0.13	1	0.87
BRI vs LAI	0.35	0.1	0.00012	0.22
EXI vs LAI	0.000013	1	0.00067	0.15
