# Supplementary material for: Stratification of hospitalized COVID-19 patients into clinical severity progression groups by immuno-phenotyping and machine learning
Source: Nat Commun. 2022 Feb 17;13:915. doi: 10.1038/s41467-022-28621-0 (PMC8854670; doi:10.1038/s41467-022-28621-0)
Supplement: Supplementary file 3 — Reporting Summary [file 41467_2022_28621_MOESM3_ESM.pdf]

nature portfolio

Corresponding author(s): Peter D. Katsikis

Last updated by author(s): January 6, 2022

Reporting Summary

Nature Portfolio wishes to improve the reproducibility of the work that we publish. This form provides structure for consistency and transparency in reporting. For further information on Nature Portfolio policies, see our Editorial Policies and the Editorial Policy Checklist.

Statistics

For all statistical analyses, confirm that the following items are present in the figure legend, table legend, main text, or Methods section.

n/a	Confirmed	
☐	☒	The exact sample size (*n*) for each experimental group/condition, given as a discrete number and unit of measurement
☐	☒	A statement on whether measurements were taken from distinct samples or whether the same sample was measured repeatedly
☐	☒	The statistical test(s) used AND whether they are one- or two-sided<br>*Only common tests should be described solely by name; describe more complex techniques in the Methods section.*
☐	☒	A description of all covariates tested
☐	☒	A description of any assumptions or corrections, such as tests of normality and adjustment for multiple comparisons
☐	☒	A full description of the statistical parameters including central tendency (e.g. means) or other basic estimates (e.g. regression coefficient) AND variation (e.g. standard deviation) or associated estimates of uncertainty (e.g. confidence intervals)
☐	☒	For null hypothesis testing, the test statistic (e.g. *F*, *t*, *r*) with confidence intervals, effect sizes, degrees of freedom and *P* value noted<br>*Give P values as exact values whenever suitable.*
☐	☒	For Bayesian analysis, information on the choice of priors and Markov chain Monte Carlo settings
☐	☒	For hierarchical and complex designs, identification of the appropriate level for tests and full reporting of outcomes
☐	☒	Estimates of effect sizes (e.g. Cohen's *d*, Pearson's *r*), indicating how they were calculated

Our web collection on statistics for biologists contains articles on many of the points above.

Software and code

Policy information about availability of computer code

Data collection	None
Data analysis	Cleaning of flow data was performed in SpectroFlo (version 2.2.0). The unsupervised, and statistical inference portions of the flow cytometry analysis were performed using OMIQ data analysis software (www.omiq.ai). We used the unsupervised analysis methods based on surface markers without any 2D gating we previously employed. The workflow included running flowCut to check for changes in channels over acquisition time, UMAP for dimensionality reduction, flowSOM for clustering, and edgeR for statistical inference.<br>Unsupervised hierarchical clustering was performed using Ward's Hierarchical Agglomerative Clustering Method (ward.d2) and optimal number of clusters were assigned with the NbClust (v1.0.12) package in R. Heatmaps were generated with R package pheatmap (v1.0.12), and Principal Component Analysis (PCA) with R function prcomp.<br>The R-code used to cluster and the statistical analysis the cytokines, antibodies and clinical data can be freely downloaded from https://bitbucket.org/immunology-emc/covid_severity_publication/src/master/.

For manuscripts utilizing custom algorithms or software that are central to the research but not yet described in published literature, software must be made available to editors and reviewers. We strongly encourage code deposition in a community repository (e.g. GitHub). See the Nature Portfolio guidelines for submitting code & software for further information.

Data

Policy information about availability of data

All manuscripts must include a data availability statement. This statement should provide the following information, where applicable:

- Accession codes, unique identifiers, or web links for publicly available datasets
- A description of any restrictions on data availability
- For clinical datasets or third party data, please ensure that the statement adheres to our policy

All figures have associated raw data. The data used in this study are available on request from the corresponding author PDK. The data are not publicly available due to participant privacy/consent.

Field-specific reporting

Please select the one below that is the best fit for your research. If you are not sure, read the appropriate sections before making your selection.

☒ Life sciences ☐ Behavioural & social sciences ☐ Ecological, evolutionary & environmental sciences

For a reference copy of the document with all sections, see nature.com/documents/nr-reporting-summary-flat.pdf

Life sciences study design

All studies must disclose on these points even when the disclosure is negative.

Sample size	Sample size was not pre-determined. Sample size was determined by available samples in both patient cohorts.
Data exclusions	Patient samples were excluded only when there was insufficient material for measurements.
Replication	Data were replicated twice by using a discovery cohort (Rotterdam cohort) and a validation cohort (Barcelona cohort). Robustness testing for clustering was also performed.
Randomization	Not relevant to the study. All patients in each cohort were analyzed as one group.
Blinding	Experimentalists and bioinformaticians were blind to clinical status or clinical measurements when measuring and analyzing cytokines and antibodies, performing and analyzing flow cytometry and when clustering patients.

Reporting for specific materials, systems and methods

We require information from authors about some types of materials, experimental systems and methods used in many studies. Here, indicate whether each material, system or method listed is relevant to your study. If you are not sure if a list item applies to your research, read the appropriate section before selecting a response.

Materials & experimental systems

n/a	Involved in the study	
☐	☒	Antibodies
☒	☐	Eukaryotic cell lines
☒	☐	Palaeontology and archaeology
☒	☐	Animals and other organisms
☐	☒	Human research participants
☐	☒	Clinical data
☒	☐	Dual use research of concern

Methods

n/a	Involved in the study	
☒	☐	ChIP-seq
☐	☒	Flow cytometry
☒	☐	MRI-based neuroimaging

Antibodies

Antibodies used

Antibody	Fluorochrome	Company	Clone	Cat#	Lot	Dilutions
Annexin V	Alexa Fluor 350	ThermoFisher Scientific	None	A23202	2092262	1:140
CD11c	eFluor 450	ThermoFisher Scientific	3.9	48-0116-42	1978160	1:56
CD123	Super Bright 436	ThermoFisher Scientific	6H6	62-1239-42	2196734	1:56
CD127	APC-R 700	BD Biosciences	HIL-7R-M21	565185	9347414	1:47
CD14	SparkBlue 550	BioLegend	63D3	367148	B305520	1:112
CD141	BB515	BD Biosciences	1A4	565084	0007679	1:56
CD159a NKG2a	APC	Miltenyi	REA110	130-113-563	5200701951	1:70
CD159c NKG2c	PE	Miltenyi	REA205	130-119-776	5200506710	1:70
CD16	BUV496	BD Biosciences	3G8	612944	0094137	1:230

CD183 CXCR3	PE-Cy7	ThermoFisher Scientific	CEW33D	25-1839-42	2082899	1:56
CD185 CXCR5	BV750	BD Biosciences	RF8B2	747111	0139400	1:116
CD19	SparkNIR 685	BioLegend	HIB19	302270	B304469	1:116
CD195 CCR5	BUV563	BD Biosciences	2D7/CCR5	741401	0192658	1:56
CD196 CCR6	BV711	BioLegend	G034E3	353436	B286323	1:116
CD197 CCR7	BV421	BioLegend	G043H7	353208	B316254	1:28
CD1c	Alexa Fluor 647	BioLegend	L161	331510	B293062	1:28
CD2	PerCP-Cy5.5	BioLegend	TS1/8	309226	B308609	1:28
CD20	Pacific Orange	ThermoFisher Scientific	HI47	MHCD2030	2204093	1:56
CD24	PE-Alexa Fluor 610	ThermoFisher Scientific	SN3	MHCD2422	2174270	1:56
CD25	PE-Alexa Fluor 700	ThermoFisher Scientific	CD25-3G10	MHCD2524	2232052	1:56
CD27	APC-H7	BD Biosciences	M-T271	560222	9113661	1:56
CD279 PD-1	BV785	BioLegend	EH12.2H7	329930	B290009	1:28
CD28	BV650	BioLegend	CD28.2	302946	B298805	1:56
CD3	BV510	BioLegend	SK7	344828	B302134	1:56
CD314	BUV615	BD Biosciences	1D11	751232	0192677	1:28
CD337	PE-Dazzle 594	BioLegend	P30-15	325232	B288706	1:28
CD38	APC-Fire 810	BioLegend	HB-7	356644	B312070	1:140
CD39	BUV661	BD Biosciences	TU66	749967	0192671	1:56
CD4	cFluor YG584	Cytek	SK3	R7-20041	20032801	1:116
CD45	PerCP	ThermoFisher Scientific	H130	MHCD4531	2194850	1:116
CD45RA	BUV395	BD Biosciences	5H9	740315	0112760	1:116
CD56	BUV737	BD Biosciences	NCAM16.2	564447	0048331	1:116
CD57	FITC	BioLegend	HNK-1	359604	B229645	1:280
CD8	BUV805	BD Biosciences	SK1	612889	0086704	1:116
CD95	PE-Cy5	ThermoFisher Scientific	DX2	15-0959-42	1919658	1:233
HLADR	PE-Fire 810	BioLegend	L243	Custom conjugated	B312582	1:233
IgD	BV480	BD Biosciences	IA6-2	566138	0036470	1:233
IgG	BV605	BD Biosciences	G18-145	563246	0009155	1:28
IgM	BV570	BioLegend	MHM-88	314517	B300089	1:56
γδTCR	PerCP-eF710	ThermoFisher Scientific	B1.1 46-	9959-42 2	172725	1:112

Validation

The 40-color spectral flow cytometry antibody panel was validated previously by Cytek Biosciences (see Park LM et al, PMID: 32830910).

Human research participants

Policy information about studies involving human research participants

Population characteristics

Patients were at least 18 years, admitted to the hospital for COVID-19 and SARS-CoV-2 genome positive by RT-PCR test. Rotterdam cohort samples were collected from patients (n=50) participating in the ConCOVID nationwide multicenter open-label randomized clinical trial in the Netherlands. Patient age was 63 (57.25-69) years (median (Q1-Q3)), and 66% were males. Patients entered the study and were sampled at a median of 2 days after hospitalization (IQR: 1, 3,75 days). Healthy control (n=14) age was 52 (41.75-62.75) years (median (Q1-Q3)) and 36% were male. The study was reviewed and approved by the institutional review board of the Erasmus University Medical Center. Written informed consent was obtained from every patient or legal representative.

Barcelona cohort samples and data from patients included in this study were provided by the Hospital Universitari Vall d'Hebron (HUVH) Biobank (PT17/0015/0047), integrated in the Spanish National Biobanks Network. The collection and secondary use of de-identified diagnostic samples and data for research was approved by the Ethics Committee for Research with Medicines of HUVH and consent was waived. Sample and data transfer to Erasmus MC for research purpose was approved by the Ethics Committee for Research with Medicines of HUVH. Patient inclusion criteria were an age of at least 18 years, having a confirmed SARS-CoV-2 genome positive by RT-PCR test and having a serum sample on the day of hospitalization. No exclusion criteria were applied. Patient age was 61 (50-70) years (median (Q1-Q3)), and 65.9% were males.

Recruitment

Patients recruited were hospitalized for COVID-19 with a positive PCR test. Patients were at least 18 years old and have samples available from the first 1 to 5 days after admission to the hospital.

Ethics oversight

Rotterdam cohort: The study was reviewed and approved by the institutional review board of the Erasmus University Medical Center.
Barcelona cohort: The collection and secondary use of de-identified diagnostic samples and data for research was approved by the Ethics Committee for Research with Medicines of University Hospital Vall d'Hebron (HUVH) and consent was waived. Sample and data transfer to Erasmus MC for research purpose was approved by the Ethics Committee for Research with Medicines of HUVH.

Note that full information on the approval of the study protocol must also be provided in the manuscript.

Clinical data

Policy information about clinical studies

All manuscripts should comply with the ICMJE guidelines for publication of clinical research and a completed CONSORT checklist must be included with all submissions.

Clinical trial registration	Not applicable. Not a clinical trial.
Study protocol	*Note where the full trial protocol can be accessed OR if not available, explain why.*
Data collection	*Describe the settings and locales of data collection, noting the time periods of recruitment and data collection.*
Outcomes	*Describe how you pre-defined primary and secondary outcome measures and how you assessed these measures.*

Flow Cytometry

Plots

Confirm that:

☒ The axis labels state the marker and fluorochrome used (e.g. CD4-FITC).

☒ The axis scales are clearly visible. Include numbers along axes only for bottom left plot of group (a 'group' is an analysis of identical markers).

☒ All plots are contour plots with outliers or pseudocolor plots.

☒ A numerical value for number of cells or percentage (with statistics) is provided.

Methodology

Sample preparation	Sample used were PBMC isolated by density centrifugation (Ficoll) from peripheral blood.
Instrument	5-laser Aurora spectral flow cytometer (Cytek Biosciences, CA)
Software	SpectroFlo (version 2.2.0), OMIQ data analysis software
Cell population abundance	One million events were collected for flow cytometry analysis. No sorting was performed.
Gating strategy	Gating strategy: Remove doublets by FCS-A vs FCS-W, exclude dead cells by annexin V stains, gate on all CD45+ cells.

☒ Tick this box to confirm that a figure exemplifying the gating strategy is provided in the Supplementary Information.
